# Supplementary material for: The MidPIC study: Midwives’ knowledge, perspectives and learning needs regarding preconception and interconception care
Source: PLoS One. 2023 Nov 9;18(11):e0289910. doi: 10.1371/journal.pone.0289910 (PMC10635450; doi:10.1371/journal.pone.0289910)
Supplement: S1 File — (DOCX) [file pone.0289910.s001.docx]

| The MidPIC study: Midwives’ knowledge, perspectives and learning needs regarding preconception and interconception care | | |
| --- | --- | --- |
| RB – Radio buttons  FT – Free text | | |
| **Part 1 Demographics** | | |
| **Gender** (RB)  1 Female  2 Male  3 Nonbinary  4 I prefer not to say | | |
| **Age** (RB)  1 Less than 24 years  2 25-34 years  3 35-44 years | | 4 45-54 years  5 55-64 years  6 65 years or older |
| **Were you born in Australia?**  1 Yes  2 No  **If ‘no’, please tell us where you were born** (FT) | |  |
| **Do you speak a language other than English at home?**  1 Yes  2 No  **If ‘yes’, please indicate what language you speak** (FT) | | |
| **Professional qualification (select all that apply)** (RB)  1 Midwife  2 Registered Nurse  3 Endorsed Midwife  4 IBCLC Lactation Consultant  5 Maternal Child Health/Child Heath qualification  6 Other professional qualifications (i.e. Paramedicine, accounting etc.) | | |
| **Did you complete your midwifery education in Australia?** (RB)  1 Yes  2 No  **If ‘no’ please indicate where you completed your midwifery education** (FT) | | |
| **Highest qualification level** (RB)  1 Bachelor’s degree  2 Graduate certificate  3 Graduate diploma  4 Masters  5 PhD | | |
| **Number of years as a midwife** (RB)  1 Less than 5 years  2 6-10 years  3 11-20 years  4 21 years or more | | |
| **State that you work in** (RB)  1 NSW  2 VIC  3 QLD  4 SA | | 5 WA  6 TAS  7 NT  9 ACT |
| **Region of place of work** (RB)  1 Metro  2 Regional  3 Rural  4 Remote | | |
| **Model of care currently employed if more than one please choose the model you predominantly work in that best describes your working arrangements** (RB)  1 Non-continuity model (standard public system)  2 Non-continuity model (standard private system)  3 Continuity model (public system, hospital-based i.e., hospital based MGP)  4 Continuity model (public system, community-based i.e., MGP connected to a birth centre)  5 Continuity model (private system/clinic, community-based i.e., endorsed midwife offering antenatal/ postnatal care)  6 Privately practicing midwife  7 Educational setting such as staff education in a hospital or education provider in university/similar  8 Research/policy/management  9 Other (FT) | | |
| **PART 2 Knowledge of pre/interconception health** | | |
| **How would you rate your overall knowledge about pre/interconception health for women?** (RB)  1 Excellent  2 Above average  3 Average  4 Below average  5 Poor  6 Nothing, I don’t know much at all | | |
| **How would you rate your knowledge about pre/interconception health for men/partners?** (RB)  1 Excellent  2 Above average  3 Average  4 Below average  5 Poor  6 Nothing, I don’t know much at all | | |
| **Please rate your knowledge in the below topics:** (For each topic, drop down option)  1 I have expert working knowledge  2 I have a good working knowledge  3 I have basic working knowledge  4 I have limited working knowledge  5 I have inadequate working knowledge  6 I have no working knowledge | | |
| **Optimising health behaviours** | Pre/interconception vitamin supplementation recommendations | |
|  | Pre/interconception vaccination recommendations | |
|  | Substance use advice for women planning pregnancy (including alcohol, smoking and other drugs) | |
|  | Reproductive impacts of hazardous living and working environments (e.g., toxin, pollutant exposure) | |
|  | Counselling on the right to autonomy, safety and satisfaction in healthy sexual relationships | |
| **Addressing pre-existing health conditions** | Potential adverse reproductive/pregnancy outcomes of diabetes mellitus | |
|  | Potential adverse reproductive/pregnancy outcomes of pre-existing hypertension | |
|  | Hemoglobulin disorders: At-risk populations and screening recommendations | |
| **Achieving a healthy weight** | Importance of healthy weight ranges for women of reproductive age | |
|  | Signs of disordered eating/eating disorders | |
|  | Dietary recommendations for women of reproductive age | |
|  | Physical activity recommendations for women of reproductive age | |
|  | Role of sleep routine in maintaining healthy weight | |
| **Optimising reproductive health** | Birth spacing recommendations | |
|  | Symptoms, screening and treatment of sexually transmitted infections | |
|  | Potential adverse reproductive outcomes of untreated sexually transmitted infections | |
|  | Stopping contraception when planning pregnancy | |
|  | Postnatal options for contraception | |
|  | Management options for unprotected sexual intercourse/unintended pregnancy | |
|  | Screening, discussion and assessment for those seeking abortion | |
| **Optimising mental health** | Awareness of challenges during periods of life course transitions (ie., parenting) and the importance of actively investing in mental health | |
|  | Sleep hygiene practices/behaviours | |
|  | Workplace rights for reproductive aged women (e.g., maternity/parental leave entitlements) | |
|  | Encouraging development and maintenance of social support networks | |
| **PART 3 Education: Learning needs related to pre/interconception health** | | |
| Which of these topics would you like to learn more about? Tick all that apply. (RB) | | |
| **Optimising health behaviours** |  Pre/interconception vitamin supplementation recommendations | |
|  |  Pre/interconception vaccination recommendations | |
|  |  Substance use advice for women planning pregnancy (including alcohol, smoking and other drugs) | |
|  |  Reproductive impacts of hazardous living and working environments (e.g., toxin, pollutant exposure) | |
|  |  Counselling on the right to autonomy, safety and satisfaction in healthy sexual relationships | |
| **Addressing pre-existing health conditions** |  Potential adverse reproductive/pregnancy outcomes of diabetes mellitus | |
|  |  Potential adverse reproductive/pregnancy outcomes of pre-existing hypertension | |
|  |  Hemoglobulin disorders: At-risk populations and screening recommendations | |
| **Achieving a healthy weight** |  Importance of healthy weight ranges for women of reproductive age | |
|  |  Signs of disordered eating/eating disorders | |
|  |  Dietary recommendations for women of reproductive age | |
|  |  Physical activity recommendations for women of reproductive age | |
| **Optimising reproductive health** |  Birth spacing recommendations | |
|  |  Symptoms, screening and treatment of sexually transmitted infections | |
|  |  Potential adverse reproductive outcomes of untreated sexually transmitted infections | |
|  |  Stopping contraception when planning pregnancy | |
|  |  Postnatal options for contraception | |
|  |  Management options for unprotected sexual intercourse/unintended pregnancy | |
|  |  Screening, discussion and assessment for those seeking abortion | |
| **Optimising mental health** |  Awareness of challenges during periods of life course transitions (ie., parenting) and the importance of actively investing in mental health | |
|  |  Sleep hygiene practices/behaviours | |
|  |  Workplace rights for reproductive aged women (e.g., maternity/parental leave entitlements) | |
|  |  Encouraging development and maintenance of social support networks | |
| **PART 3.1 Education: Learning preferences** | | |
| **How would you prefer this education to be delivered? Please select top three preferences.** (RB)  1 Online e-learning  2 Face-to-face training within workplaces  3 Face-to-face training external to workplaces  4 Online webinars  5 Postgraduate certificate with qualification  6 Micro-credentialled 1 day workshops that lead to a Grad Cert  7 Podcasts | | |
| **PART 4 Attitudes towards pre/interconception health** | | |
| **Please indicate how much you agree with the following statements:**  (RB - *Strongly agree, agree, disagree, strongly disagree)* | | |
| Providing pre/interconception care is within the midwifery scope of practice. | | |
| Midwives are well placed to be the primary providers of pre/interconception care. | | |
| Midwives have a role in promoting the pre/interconception health of men and partners. | | |
| In my practice, I often encounter health states that could/should have been managed before pregnancy. (i.e., anaemia, MMR immunisation, sexual health) | | |
| Planning to conceive is a personal decision and should only be discussed when initiated by the woman/client. | | |
| Pre/interconception care should be provided for all people of reproductive age, not only those actively planning pregnancy. | | |
| **PART 5 Service planning** | | |
| **What do you think are the top 3 barriers to establishing midwives’ role in pre/interconception care in Australia?** (RB – 3 options)  1 Not prioritised by women/people/service users  2 Not prioritised by healthcare professionals  3 Not prioritised in service planning/budgeting  4 Lack of education/educational opportunities  5 Time and staffing constraints  6 Specialised services not available  7 Lack of available evidence based guidelines  8 Uncertainty around which profession is responsible  9 Other | | |
| **Are you already working in a setting where you provide preconception/interconception care?** (RB)  1 I already provide pre/interconception care  2 I could provide pre/interconception care but don’t  3 I do not provide pre/interconception care but I would like to  **4 I do not provide pre/interconception care and I’m not interested in it** | | |
| **If it was possible and relevant to your current setting, would you be interested in providing pre/interconception care and education more regularly in your current role as a midwife?** (RB)  1 Yes  2 No  3 Unsure | | |
| **If there was an option the work in a community setting as a midwife providing preconception care, would this interest you?** (RB)  1 Yes  2 No  3 Unsure | | |
| **Do you have any further comments you’d like to make about midwives’ providing preconception and interconception care in Australia** | | |
